# A Trend Analysis of Metastatic Lung Cancer Disparities in the United States in the Era of Lung Cancer Screening

**DOI:** 10.7759/cureus.85184

**Published:** 2025-06-01

**Authors:** Maxwell Akanbi, Orimisan S Adekolujo, Ling Wang, Daniel Isaac, Jerry Kenmoe, Ahsan Wahab, Oyebimpe Adekolujo, Borys Hrinczenko

**Affiliations:** 1 Hematology and Medical Oncology, McLaren Greater Lansing, East Lansing, USA; 2 Internal Medicine, McLaren Flint, Michigan State University College of Human Medicine, Flint, USA; 3 Medicine, Michigan State University, East Lansing, USA; 4 Hematology and Oncology, Michigan State University-Breslin Cancer Center, Lansing, USA; 5 Hematology and Medical Oncology, University of Illinois at Chicago, Chicago, USA

**Keywords:** epidemilogy, incidence, lung cancer, screening, seer database

## Abstract

Background: The introduction of low-dose chest computed tomography (LDCT) screening has shown promise in improving early detection and reducing lung cancer mortality. Nonetheless, marginalized communities have displayed limited participation in lung cancer screening, potentially diminishing its advantages within these populations. We assessed the trends in metastatic lung cancer incidence and evaluated disparities by sex, race/ethnicity, and residence type in the United States.

Methods: We analyzed data from 445,715 adults aged between 55 and 80 years with metastatic lung cancer in the Surveillance, Epidemiology, and End Results (SEER) database from 2004 to 2019. We generated age-adjusted incidence rates (AAIR) and used Joinpoint regression models to estimate the average annual percentage change (AAPC) in incidence across different subgroups. We employed the Joinpoint test of parallelism to identify differences in metastatic lung cancer incidence by sex, race/ethnicity, and residence.

Results: The incidence of metastatic lung cancer declined more rapidly from 2014 to 2019, compared with 2004 to 2014, in men (AAPC 5.5% (2014 to 2019), 2.3% (2004-2014)) and women (1.7% (2014-2019), 0.86% (2004-2014)), with the test of parallelism indicating narrowing of disparities from 2014 to 2019. Similarly, metastatic lung cancer incidence declined more rapidly from 2014 to 2016 in metropolitan and non-metropolitan residents, with parallel trend lines. However, among racial groups, only White and Black patients had a significant reduction in metastatic lung cancer incidence from 2014 to 2019.

Conclusions: Our analysis revealed a significant decline in metastatic lung cancer incidence in most groups since 2014, coinciding with the implementation of LDCT screening. Non-Hispanic American Indian/Alaskan, non-Hispanic Asian/Pacific Islander, and Hispanic patients, however, showed no significant trend change during the study period.

## Introduction

Lung cancer is the leading cause of cancer mortality in the United States (US) [1]. In 2024, lung cancer was responsible for 20% of cancer-related deaths in men and 21% in women in the US [1]. Late diagnosis significantly contributes to the high mortality among individuals with lung cancer [2]. Among patients with lung cancer from 2012 to 2018 in the Surveillance, Epidemiology, and End Results (SEER) Program database, 77% had regional or distant metastasis at the time of diagnosis [3]. The five-year relative survival for lung cancer is 7% for distant metastasis, 34% for regional metastasis, and 61% for localized disease [3]. Therefore, timely lung cancer diagnosis is crucial for reducing lung cancer mortality.

The National Lung Screening Trial (NLST) was the first study to demonstrate the survival benefits of the low-dose chest computerized tomography scan (LDCT) for lung cancer [4]. The study showed a 20% relative reduction in mortality from lung cancer with LDCT screening compared with chest radiography in high-risk populations [4]. In response to the results of the NLST, the US Preventive Services Task Force (USPSTF) in 2013 recommended annual lung cancer screening with LDCT in adults aged between 55 and 80 years with a 30-pack-year smoking history and who currently smoke or have quit within the past 15 years [5]. Reports indicate, however, that the uptake of lung cancer screening has been low across the US, particularly among high-risk and minority populations [6,7]. Disparities in lung cancer screening could widen historical disparities in lung cancer survival.

Beginning in 2014, the US has experienced a more rapid decline in the incidence of metastatic lung cancer, with a corresponding increase in the incidence of local lung cancer, indicating earlier lung cancer diagnosis [8]. The timing of this stage shift strongly suggests that earlier lung cancer diagnoses may have resulted from the USPSTF lung cancer screening recommendations. Increased access to care through the Patient Protection and Affordable Care Act (ACA), implemented in 2014, may also have contributed to the stage shift for lung cancer by improving access to lung cancer screening. While there has been a more rapid decline in the incidence of metastatic lung cancer, it is unclear if disparities exist among vulnerable groups. To address these concerns, we conducted a descriptive analysis of trends in metastatic lung cancer by sex, race/ethnicity, and residence in the US general population, using nationally representative data from the SEER database.

## Materials and methods

Databases

We extracted data from the Incidence-SEER Research Plus Limited-Field Data, 22 Registries, Nov 2021 Sub (2000-2019) release in April 2022 (www.seer.cancer.gov). SEER 22 covers approximately 47.9 % of the U.S. population (based on the 2010 census). The SEER database classifies cancer histology and topography information based on the third edition of the International Classification of Diseases for Oncology (ICD-O-3/WHO 2008) [3].

For this study, we appropriated data from patients aged between 55 and 80 years with cancer of the lung or bronchus (based on the ICD-03/WHO 2008 classification) with lung cancer at the time of cancer detection. We identified lung cancer cases using ICD-10 codes C340-349 [9]. Based on the SEER 'Summary/History Stage,' lung cancer was staged as localized, regional, or distant. For this study, we used data from adults classified as having 'distant' lung cancer at diagnosis. (We used metastatic lung cancer to describe the 'distant' stage). Since this staging was only available from 2004, our analytic sample included individuals diagnosed with lung cancer from 2004 to 2019. We excluded data from 2020 to 2022 (most recent SEER data) due to the confounding effect of the coronavirus pandemic.

We obtained annual age-adjusted incidence rates (AAIR) of mLC per 100,000 person-years, stratified by sex (male or female), race/ethnicity (White, Black, Non-Hispanic American Indian/Alaskan Native (NHAI/AN), Non-Hispanic Asian/Pacific Islander (NHAPI), or Hispanic), and residence type (metropolitan or non-metropolitan). We extracted race/ethnicity data from the 'Race and Origin Recode' database and the residence data from the 'County Attributes/Rural-Urban Continuum' database. Age adjustment for cancer incidence rates in SEER was computed using the 2000 US standard population [10]. We obtained ethical approval for the study from the Institutional Review Board of McLaren Hospital, Flint, MI (approval number: 202204-06).

Statistical analysis

We used Joinpoint regression models to analyze trends of AAIR of metastatic lung cancer by sex (female, male), race/ethnicity (White, Black, NHAI/AN), NHAPI, and Hispanic), and residence (metropolitan, non-metropolitan). The joinpoint model uses two fit indices (i.e., Akaike information criterion (AIC) and Bayesian information criterion (BIC)) to determine when and how often the annual percentage change (APC) changes (number of joinpoints). APC assumes a constant percentage change every year on a log scale. APC assumes a constant percentage change every year on a log scale. The APC within two adjacent joinpoints is calculated as APC=(e^β^ -1 x100). The coefficient (β) is the estimated average change (slope) obtained from linear regression: log (y) = α + βw. (y is the age-adjusted incidence of metastatic lung cancer rate, w is the number of years within two adjacent joinpoints, and β captures the fixed effect of time on the age-adjusted incidence of metastatic lung cancer rates). We placed no limitations on the number of joinpoints.

We compared metastatic lung cancer trends by sex (male versus female), race/ethnicity (White patients versus Black patients), and residence (metropolitan versus non-metropolitan) using the Joinpoint trend parallelism test [11]. We excluded other racial/ethnic groups from the comparative analysis because they did not exhibit any joinpoints throughout the study period. The absence of joinpoints limited our ability to assess shifts in incidence patterns over time within these groups, making direct comparisons with populations that demonstrated distinct changes in trend less meaningful.

We conducted statistical analyses using the Surveillance Research Program, National Cancer Institute SEER*Stat software (www.seer.cancer.gov/seerstat) version 8.4.0, and the National Cancer Institute Joinpoint regression program (Joinpoint Regression Program, Version 4.9.1.0 - April 2022; Statistical Methodology and Applications Branch, Surveillance Research Program, National Cancer Institute).

## Results

Patient characteristics

We analyzed data from 445,715 patients with metastatic lung cancer; 55% (n=247,262) were male, and 76 % (n=340,407) were White. The majority (84%) were from metropolitan counties. The overall incidence of metastatic lung cancer from 2004 to 2019 was 87.7 (95% CI 87.4-87.9) per 100,000 person-years. We summarized the characteristics of the population in Table 1. 

**Table 1:** Characteristics of adults with distant cancer of the lung or bronchus 2004-2019 (SEER database) SEER: Surveillance, Epidemiology, and End Results; Data include patients aged between 55 and 80 years at the time of lung cancer diagnosis

Parameters	Total metastatic lung cancer cases reported in SEER (N= 445,715 Count (%))
Sex, n (%)	
Male	247,262 (55)
Female	198,453 (45)
Age, years, mean (SD)	68 (7.0)
Race/ethnicity, n (%)	
White	340,407 (76.4)
Black	51,793 (11.6)
Non-Hispanic American Indian/Alaska Native	1,797 (0.4)
Non-Hispanic Asian or Pacific Islander	22,007 (4.9)
Hispanic (all races)	29,308 (6.6)
Non-Hispanic Unknown	403 (0.1)
Residence, n (%)	
Metropolitan counties	375,332 (84.2)
Non-metropolitan counties	69,964 (13.2)
Unknown/Missing/No match (Alaska or Hawaii-entire state)	398 (0.09)
Unknown/Missing/No match	21 (0.00)
Median household income inflation (adjusted to 2019, n (%))	
< $35,000	6,859 (1.5)
$35,000- $54,999	119,711 (26.9)
$55,000 – $74,999	204,000 (45.8)
$75,000+	115,124 (25.8)
Unknown/Missing	21 (0.0)

Metastatic lung cancer incidence trends based on sex stratification

**Figure 1:**
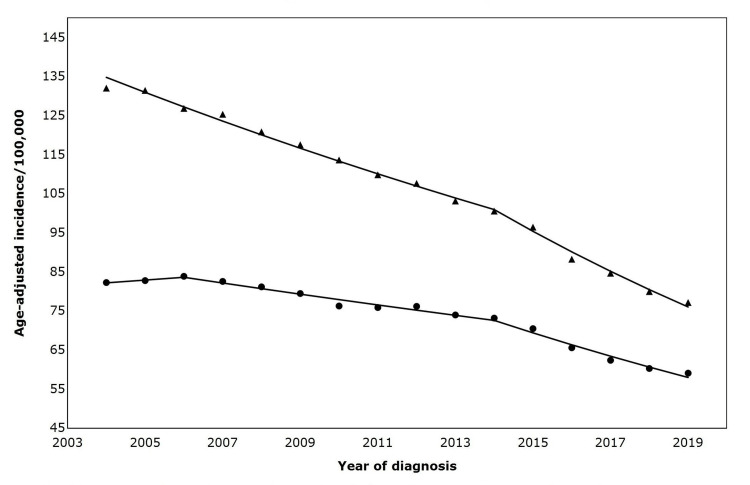
Age-adjusted incidence of metastatic lung cancer by sex (2004-2019) Symbols: ▲= Male; ⬤ = Female Trends in annual percent change (APC) by sex are as follows: Males: 2004–2014: APC = –2.85 (p < 0.05); 2014–2019: APC = –5.51 (p < 0.05); and Females: 2004–2006: APC = +0.85 (not statistically significant); 2006–2014: APC = –1.70 (p < 0.05); 2014–2019: APC = –4.40 (p < 0.05). Trend comparison: Parallelism test: rejected, APC trends not parallel between males and females

From 2004 to 2019, the incidence of metastatic lung cancer was higher in men than in women. The overall incidence rate in men was 133.1 (95% CI 130.3 - 134.0) per 100,000 person-years, while in women it was 82.3 (95% CI 81.0-83.7) per 100,000 person-years. The incidence of metastatic lung cancer in men reduced at an average annual percentage change (AAPC) of 2.3% from 2004 to 2014, with a more rapid decline of 5.5% from 2014 to 2014 (Table 2). In contrast, among women, there was an initial increase in metastatic lung cancer incidence from 2004 to 2006 (AAPC, 0.86%), followed by a decline (AAPC, 1.7%) from 2006 to 2014. Notably, from 2014 to 2019, women experienced a significant decrease in the incidence of metastatic lung cancer compared to the previous period (Figure 1).

**Table 2:** Temporal trends of metastatic lung cancer incidence in the United States (SEER Research Plus Limited-Field Data, 22 Registries), 2004-2019 APC: annual percent change; NHAI/AN: Non-Hispanic American Indians and Alaska Native, NHAPI: Non-Hispanic Asian and Pacific Islander; SEER: Surveillance, Epidemiology, and End Results

Variable	Trend 1			Trend 2			Trend 3		
	Year	APC (95% CI)	P-value	Year	APC (95% CI)	P-value	Year	APC (95% CI)	P-value
Sex									
Male	20004-2014	-2.9 (-3.1, -2.6)	<0.001	2014-2019	-5.5 (-6.3, -4.7)	<0.001	-	-	-
Female	2004-2006	0.86 (-4.1, 6.1)	0.71	2006-2014	-1.8 (-2.4, -1.1)	<0.001	2014-2019	-4.4 (-5.5, -3.3)	<0.001
Race/Ethnicity									
White	2004-2007	-0.6 (-2.1, 0.9)	0.408	2007-2014	-2.3 (-2.8, -1.8)	<0.001	2014-2019	-4.8 (-5.4, -4.1)	<0.001
Black	2004-2014	-1.9 (-1.2, -1.3)	<0.001	2014-2019	-5.6 (-7.1, -4.0)	<0.001	-	-	-
NHAI/AN	2004-2019	-2.2 (-3.1, -1.2)	<0.001	-	-	-	-	-	-
NHAPI	2014-2019	-1.8 (-2.2, -1.4)	<0.01	-	-	-	-	-	-
Hispanic (all races)	2004-2019	-3.3 (-3.7, -1.4)	<0.001	-	-	-	-	-	-
Residence									
Metropolitan counties	2004-2007	-1.3 (-3.1, 0.6)	0.147	2007-2014	-2.8 (-3.4, -2.2)	<0.001	2014-2019	-5.0 (-5.9, -4.2)	<0.001
Non-metropolitan counties	2004-2015	-0.8 (-1.2, -0.4)	0.001	2015-2019	-4.6 (-6.4, -2.8)	<0.001	-	-	-

The Joinpoint parallelism test results showed that the overall trends in metastatic lung cancer incidence between men and women were not parallel (AAPC difference -1.4 (95% CI -2.2, -0.7), p=0.0). In sub-segment analyses, on average, metastatic lung cancer incidence declined by 1.6% slower in women than men from 2004 to 2014 (AAPC difference -1.6, (95% CI -2.6, -0.6, p=0.0). However, from 2014 to 2019, men's and women's metastatic lung cancer trends did not significantly differ (AAPC difference -1.1 (95% CI -2.3, 0.0), p=0.1).

Metastatic lung cancer incidence trends based on race/ethnic stratification

The incidence of metastatic lung cancer was highest among Black patients and lowest among Hispanic patients. NHAI/AN, NHAPI, and Hispanic patients experienced no joinpoints throughout the study period (2004 to 2019), and their AAPCs were 1.8%, 2.16%, and 3.3%, respectively.

**Figure 2:**
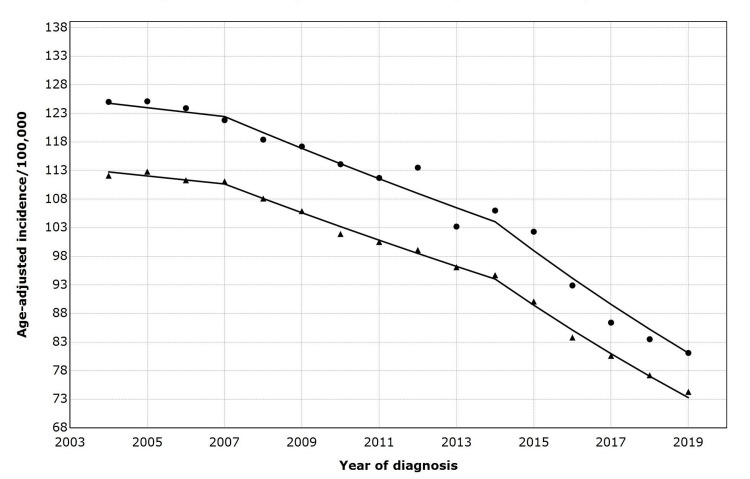
Age-adjusted incidence of metastatic lung cancer by race/ethnicity (2004-2019) Symbols: ▲= White patients; ⬤ = Black patients Trends in annual percentage change (APC) by race/ethnicity are as follows: 2004–2007: APC = –0.63 (not statistically significant); 2007–2014: APC = –2.30 (p < 0.05); 2014–2019: APC = –4.86 (p < 0.05) Parallelism test: not rejected, APC trends parallel between White and Black patients.

Among Black patients, metastatic lung cancer incidence declined by an AAPC of 1.8 % from 2004 to 2014 and more rapidly (AAPC 5.6 %) from 2014 to 2019. For White patients, the incidence declined by an AAPC of 0.6% between 2004 and 2007, then a more rapid decline of 2.3% between 2007 and 2014. Between 2014 and 2019, White patients experienced the most significant reduction in the incidence of metastatic lung cancer from 2004 to 2019 (Figure 2).

As we identified joinpoints exclusively in Black and White patients, we performed a Joinpoint parallelism test to compare metastatic lung cancer trends between the groups. The results in Table 3 indicate no statistically significant difference in metastatic lung cancer trends between Black and White patients from 2004 to 2019.

**Table 3:** Comparison of temporal trends of metastatic lung cancer incidence in the United States (SEER Research Plus Limited-Field Data, 22 Registries), 2004-2019 Results obtained from the joinpoint parallelism test; **Similar results were obtained when the joinpoint was in 2015. APC: annual percent change; NHW: Non-Hispanic White; NHB: Non-Hispanic Black; SEER: Surveillance, Epidemiology, and End Results

Variables	Period	APC difference (95% CI)	P-value
Sex			
Male versus female	20004-2014	-1.6 (-2.6,-0.6)	0.0
	2014-2019	-1.1 (-2.3, 0.0)	0.1
Race/Ethnicity			
NHW versus NHB	2004-2014	0.1 (-0.6, 0.8)	0.8
	2014-2019	0.8 (-0.7, 2.3)	0.3
Residence			
Metropolitan counties versus non-metropolitan counties**	2004-2014	-1.5 (-2.2, -0.9)	0.0
	2014-2019	-1.2 (-2.6, 0.3)	0.1

Metastatic lung cancer incidence trends based on residential stratification

**Figure 3:**
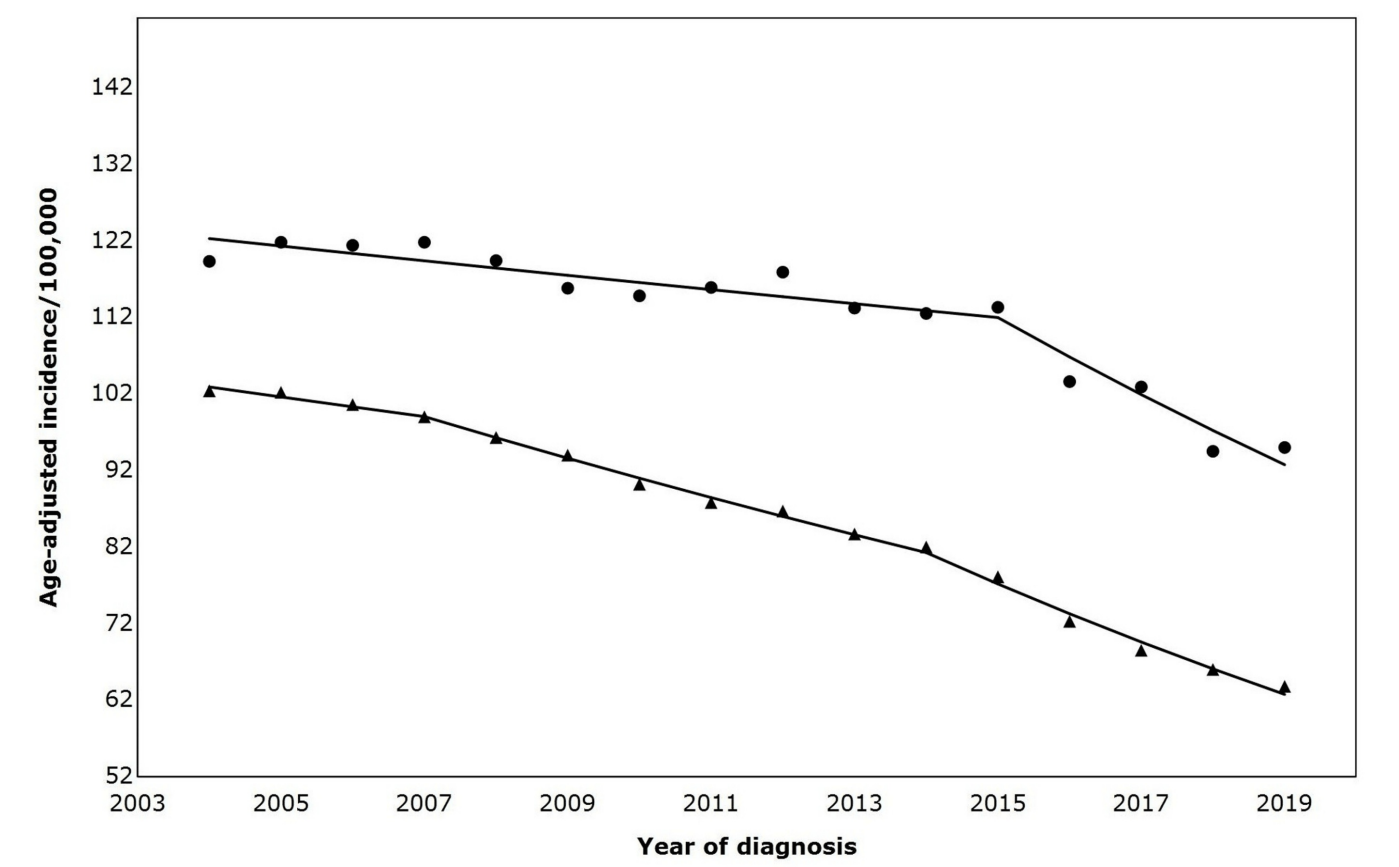
Age-adjusted incidence of advanced lung in the United States (2004-2019), by residence Symbols: ▲= Metropolitan; ⬤ = Non-Metropolitan Trends in annual percent change (APC) in metropolitan vs. non-metropolitan areas are as follows: Metropolitan areas: 2004–2007: APC = –1.27 (not statistically significant); 2007–2014: APC = –2.78 (p < 0.05); 2014–2019: APC = –5.04 (p < 0.05); Non-metropolitan areas: 2004–2015: APC = –0.8 (p < 0.05); 2015–2019: APC = –4.6 (p < 0.05) Trend comparison: Parallelism test: rejected, APC trends not parallel between metropolitan and non-metropolitan regions.

From 2004 to 2019, the incidence of metastatic lung cancer was higher among non-metropolitan than metropolitan residents. In the non-metropolitan population, the incidence of metastatic lung cancer declined by an AAPC by 0.8% from 2004 to 2015 and decreased more rapidly (AAPC 4.6%) from 2015 to 2019. Among metropolitan residents, there were two joinpoints (2007 and 2014), and metastatic lung cancer incidence declined by 1.3%, 2.8%, and 5.0% for 2004 - 2007, 2007 - 2014, and 2014 - 2019, respectively (Figure 3). The Joinpoint test of parallelism showed no significant difference in incident metastatic lung cancer trends by residence (Table 3).

## Discussion

We describe trends in the incidence of metastatic lung cancer among adults aged between 55 and 80 years in the US general population in the context of the 2013 USPSTF lung cancer screening guidelines. We also investigated disparities in the incidence of metastatic lung cancer in minority populations following the introduction of lung cancer screening in the US [8]. We found that most minority groups experienced an accelerated decline in the incidence of metastatic lung cancer in the years following the introduction of lung cancer screening. However, among racial/ethnic minority groups, only Black patients experienced a more rapid decline in the incidence of metastatic lung cancer since the introduction of lung cancer screening in the US.

Race has been shown to influence lung cancer epidemiology and access to lung cancer screening in the US [12]. Black patients develop lung cancer at younger ages and with lower pack-years of smoking than White patients [13]. Because of this, various studies predicted that the 2013 USTF lung cancer screening guidelines might be less sensitive among Black patients [14]. However, we observed that since 2014, the incidence of metastatic lung cancer has decreased rapidly among both Black and White patients, showing similar trends for both groups. This finding is significant because Black and White patients have significantly higher metastatic lung cancer incidence than other ethnic/racial groups [15]. 

Unlike Black and White patients, Hispanic patients, NHAI/AN patients, and NHAPI patients experienced no change in the trend of metastatic lung cancer from 2004 to 2019. While these groups have a relatively lower incidence of lung cancer than Black and White patients, lung cancer is a significant cause of morbidity and mortality in some of these populations [16]. In 2017, cancer was the leading cause of mortality among NHAPI patients, accounting for 25.5% of deaths in women and 24.8% in men, with lung cancer being the leading cause of cancer death [17]. Among Hispanic patients, lung cancer is the leading cause of cancer-related mortality [18]. Like Black patients, these populations are underrepresented in current lung cancer screening studies, and the optimal screening strategy for these populations remains uncertain [4, 12]. Also, these are a diverse group of people with different characteristics and will be better served with dedicated studies to evaluate cancer epidemiology among the unique populations.

Our results indicate that the 2013 USPSTF guidelines may have enhanced earlier lung cancer diagnosis in women. While the incidence of metastatic lung cancer declined more rapidly in men than in women from 2004 to 2014, our results indicate that this disparity was eliminated from 2014 to 2019. Yet there are genuine concerns regarding the sensitivity of the 2013 USPSTF in aiding early lung cancer detection among women [19]. The current lung cancer screening guidelines prioritize screening heavy smokers, but non-smokers who develop lung cancer are nearly twice as likely to be women as men [20]. Also, women develop tobacco-related lung cancer with lower smoking exposure than men, indicating that lower tobacco exposure thresholds could increase the sensitivity of lung cancer screening in women [21]. Given these factors that make lung cancer screening guidelines less sensitive in women, it is significant that we found similar declines in the incidence of metastatic lung cancer in men and women following the implementation of the lung cancer screening guidelines in the US. However, lung cancer remains a significant cause of mortality among women [15], and developing screening algorithms that can equitably identify women at high risk for lung cancer should be a priority.

We found that overall (2004-2019), the incidence of metastatic lung cancer declined more rapidly among metropolitan residents, with two joinpoints occurring in 2007 and 2014. While the incidence of metastatic lung cancer started declining more rapidly in 2014 in the metropolitan population, the non-metropolitan population did not experience a change in the incidence of metastatic lung cancer until 2015. These results suggest a delayed uptake of lung cancer screening in non-metropolitan communities, which may widen disparities in lung cancer outcomes based on residence. Previous studies have reported that facilities for LDCT for lung cancer screening are limited in rural communities across the US, which may restrict access to lung cancer screening [22]. Reducing the burden of lung cancer in rural communities is crucial and requires a multifaceted approach. The prevalence and intensity of cigarette smoking are higher in rural communities, and individuals in rural communities initiate cigarette smoking at a younger age, coupled with socioeconomic deprivation, has resulted in higher lung cancer incidence and mortality in rural communities in the US [15,23,24]. Initiatives to improve access to and utilization of lung cancer screening in rural communities must be coupled with effective interventions to reduce cigarette smoking.

Our study has some limitations. First, it is an observational study, in which unmeasured confounding factors may influence. In addition, our study may be impacted by some limitations inherent in the SEER database, such as variations in coding, misclassification, reporting, and migration of patients in and out of the SEER registry area [25]. Also, while we would prefer a more extended follow-up period, the disruption of health services caused by COVID-19 would be a significant confounder when evaluating trends from 2020. Lastly, while we reported on trends of all histologic types of lung cancer, we know that some studies have reported differences in cancer trends based on histologic types, but that was not the focus of our study [26]. Despite these limitations, our study has numerous strengths. Using the SEER database, which covers about 48% of the US population, we provided external validation of the benefits of LDCT screening demonstrated in highly regulated clinical trials. We also examined changes in the incidence of metastatic lung cancer in marginalized subgroups of the population to determine whether the benefits of the intervention varied by sex, race, or residence [27]. Oversampling minority populations in the SEER database strengthened the validity of our results in these sub-populations [28].

## Conclusions

In conclusion, this study highlights the significant impact of the 2013 USPSTF lung cancer screening guidelines on reducing metastatic lung cancer incidence, particularly among Black and White populations. However, persistent disparities exist for other racial/ethnic groups, including Hispanic, American Indian/Alaska Native, and Asian or Pacific Islander populations, who did not experience similar declines. These findings underscore the need for more inclusive screening criteria and targeted outreach to ensure equitable benefits across all populations. Additionally, the delayed decline in metastatic cases among rural communities suggests barriers to screening access in non-metropolitan areas, calling for expanded healthcare infrastructure and smoking cessation programs to address geographic disparities.

The study also demonstrates that while lung cancer screening has contributed to earlier detection and reduced metastatic disease in both men and women, further refinements to screening guidelines may be necessary to improve sensitivity for women and non-smokers. Given that lung cancer remains a leading cause of cancer-related mortality in underrepresented groups, future research should focus on optimizing risk assessment models and increasing participation in screening programs. Addressing these gaps through policy changes, community engagement, and tailored interventions will be essential to achieving equitable reductions in lung cancer burden across all demographic and geographic populations.
